# Maximizing the Potential of Longitudinal Cohorts for Research in Neurodegenerative Diseases: A Community Perspective

**DOI:** 10.3389/fnins.2017.00467

**Published:** 2017-08-29

**Authors:** Catherine J. Moody, Derick Mitchell, Grace Kiser, Dag Aarsland, Daniela Berg, Carol Brayne, Alberto Costa, Mohammad A. Ikram, Gail Mountain, Jonathan D. Rohrer, Charlotte E. Teunissen, Leonard H. van den Berg, Joanna M. Wardlaw

**Affiliations:** ^1^Medical Research Council Head Office London, United Kingdom; ^2^Irish Platform for Patient Organisations, Science and Industry Dublin, Ireland; ^3^EU Joint Programme – Neurodegenerative Disease Research Paris, France; ^4^Centre for Age-Related Medicine, Stavanger University Hospital Stavanger, Norway; ^5^Department of Old Age Psychiatry, Institute of Psychiatry, Psychology, and Neuroscience, King's College London London, United Kingdom; ^6^Department of Neurology, Christian-Albrechts-University of Kiel Kiel, Germany; ^7^Hertie-Institute of Clinical Brain Research Tübingen, Germany; ^8^Cambridge Institute of Public Health, University of Cambridge Cambridge, United Kingdom; ^9^IRCCS Fondazione Santa Lucia Rome, Italy; ^10^Università degli Studi Niccolò Cusano Rome, Italy; ^11^Erasmus University Medical Centre Rotterdam, Netherlands; ^12^School of Health and Related Research, University of Sheffield Sheffield, United Kingdom; ^13^Dementia Research Centre, University College London Institute of Neurology London, United Kingdom; ^14^VU University Medical Centre Amsterdam, Netherlands; ^15^Department of Neurology and Neurosurgery, Brain Center Rudolf Magnus, University Medical Center Utrecht Utrecht, Netherlands; ^16^Centre for Clinical Brain Sciences, UK Dementia Research Institute at the University of Edinburgh Edinburgh, United Kingdom

**Keywords:** longitudinal cohort studies, research, transnational working groups, flexible funding mechanism, joint programming, neurodegenerative disease

## Abstract

Despite a wealth of activity across the globe in the area of longitudinal population cohorts, surprisingly little information is available on the natural biomedical history of a number of age-related neurodegenerative diseases (ND), and the scope for intervention studies based on these cohorts is only just beginning to be explored. The Joint Programming Initiative on Neurodegenerative Disease Research (JPND) recently developed a novel funding mechanism to rapidly mobilize scientists to address these issues from a broad, international community perspective. Ten expert Working Groups, bringing together a diverse range of community members and covering a wide ND landscape [Alzheimer's, Parkinson's, frontotemporal degeneration, amyotrophic lateral sclerosis (ALS), Lewy-body and vascular dementia] were formed to discuss and propose potential approaches to better exploiting and coordinating cohort studies. The purpose of this work is to highlight the novel funding process along with a broad overview of the guidelines and recommendations generated by the ten groups, which include investigations into multiple methodologies such as cognition/functional assessment, biomarkers and biobanking, imaging, health and social outcomes, and pre-symptomatic ND. All of these were published in reports that are now publicly available online.

## Introduction

Neurodegenerative diseases (ND) are debilitating conditions that affect neurons, primarily in the human brain and, in some cases, the spinal cord. Neurons are typically unable to reproduce or repair themselves, so when they become damaged or die they cannot be replaced by the body. There are currently no treatments for ND, which result in the progressive degeneration and/or death of nerve cells. This causes problems with movement (called hypo-/bradykinesia, tremor or ataxias), muscle strength (called motor neuron diseases) or mental functioning (called dementias). Dementias are responsible for one of the greatest challenges facing the world's health and social care systems, with Alzheimer's disease (AD) representing approximately 60–70% of all dementia cases.

The Joint Programming Initiative on Neurodegenerative Disease Research (JPND) brings together 30 countries to accelerate research progress in the ND field by defragmenting and aligning national investments and research agendas. Since its first call for proposals in 2011, JPND has raised more than €100 million of new money from national budgets of participating counties and has supported more than 70 trans-national research projects from EU member (and associated) states and partner countries such as Canada, Switzerland and Australia.

In 2014, JPND created a novel funding mechanism to support ten new, international expert Working Groups focused on rapidly building consensus around some of the major challenges facing the use of cohorts for ND research. The purpose of this paper is to present this funding process along with a broad overview of the guidelines and recommendations generated by the ten groups, all of which are now publicly available in their full version online. Each Working Group addressed different issues that contribute to cohorts being under-used for ND research. We hope that readers considering the use of cohort studies for ND research will be interested to find out more by going to the relevant reports and full publications.

### Longitudinal cohort studies in ND research

Longitudinal cohort studies, which gather data on populations over time in order to establish correlations, are widely acknowledged as important resources for multi-disciplinary ND research into the causes and progression of disease (e.g., determining risk factors). Linking and comparing such studies together would allow researchers to draw broader and statistically more powerful conclusions that could lead to a far deeper understanding of disease. Yet significant methodological variations across cohort studies, including in data collection, measurement, and analysis, impede such linkages. In short, cohort studies are not yet able to be fully leveraged for the needs of ND research, with the potential for more to be done to promote (i) untapped research opportunities they might provide, (ii) best practices in data collection/analysis, and (iii) accessibility of study data.

In 2013, JPND published a report^1^ identifying gaps and cases for new activity in areas of unmet need and outlining how to add value to existing cohort investments. The report demonstrated that despite a wealth of cohort activity across Europe, surprisingly little information is available on the natural biomedical history of ND, and the scope for intervention studies based on these cohorts is only just beginning to be explored. Given the opportunities offered by the recent evidence for convergence amongst risk factors and underlying pathologies across ND, it appears essential that steps are taken to improve and coordinate existing capabilities. Examples include the links between immune system disruption and AD, protein aggregation underlying AD, PD and prion disease, the commonality in C9orf72 hexanucleotide repeat expansions between forms of frontotemporal degeneration (FTD) and amyotrophic lateral sclerosis (ALS) and the high prevalence of vascular disease that includes risk factors and clinical expression of AD (Goedert, 2015; Rohrer et al., 2015; Colonna and Wang, 2016).

### A novel, flexible funding mechanism to accelerate progress

Based on the recommendations from the 2013 report, JPND began developing actions to produce the methodological and technical solutions required to catalyze progress in this area, both to promote better use of existing data across studies, and to provide a more interoperable approach to future data collection.

Specifically, JPND devised a new funding process called a “rapid action” call to bring together key leaders in the field to discuss and propose potential solutions to the barriers holding up progress. This call, launched in April 2014, established Working Groups, made up of a diverse range of community members, to collaborate across national and disciplinary borders with the ultimate aim of producing methodologies and frameworks that could help the international scientific community optimize the use of cohort studies for ND research. This could be done either by providing a framework for exploiting and harmonizing existing or planned cohort studies and/or as a basis for developing new research proposals.

Working Groups were identified by JPND on a competitive basis, with each group receiving up to €50 k to enable it to deliver its work within 6 months. Twenty-two applications were received, of which ten were recommended for funding by the review panel; the successful applications, comprising some 200 researchers, represent a diverse range of ND and methodologies, as summarized in Table 1. Indeed, members of the Working Groups go beyond the 30 JPND member countries and include experts based in several additional countries, including China, Russia, Singapore and the USA.

**Table 1:** List of ten funded Working Groups.

**Working group**	**Coordinator and sponsor country**	**Number of countries**
HD-READy (High-Dimensional Research in Alzheimer's Disease)	M. Arfan Ikram, Erasmus University Medical Centre, Rotterdam, Netherlands Sponsor country: Netherlands	Eight
Harmonization and innovation of cognitive, behavioral and functional assessment in neurodegenerative dementias	Alberto Costa, IRCCS Fondazione Santa Lucia, Rome, Italy Sponsor country: Italy	Nine
NETCALS (Network of Cohort Assessment in ALS)	Leonard van den Berg, University Medical Centre Utrecht, Utrecht, Netherlands Sponsor country: France	Twelve
21st Century Eurodem—Repurposing Cohorts for Dementia Studies	Carol Brayne, University of Cambridge, UK Sponsor country: Sweden	Ten
Multi-center cohort-studies in Lewy-body dementia: Challenges in harmonizing different clinical and biomarker protocols	Dag Aarsland, Stavanger University Hospital, Stavanger, Norway Sponsor country: Norway	Nine
Presymptomatic Neurodegeneration Initiative (PreNI): Developing a methodological framework for trials in presymptomatic neurodegenerative disease	Jonathan Rohrer, University College London, UK Sponsor country: UK	Six
Harmonization of biomarker assessment in longitudinal cohort studies in Parkinson's disease	Daniela Berg, Hertie-Institute for Clinical Brain Research and German Center for Neurodegenerative Diseases, Tübingen, Germany Sponsor country: Germany	Seven
Dementia Outcome Measures: Charting New Territory	Gail Mountain, University of Sheffield, UK Sponsor country: Denmark	Five
Body fluid biobanking of longitudinal cohorts in neurodegenerative diseases	Charlotte Teunissen, VU University Medical Centre, Amsterdam, Netherlands Sponsor country: Luxembourg	Five
METACOHORTS: Realizing the potential of cohort studies to determine the vascular contribution to neurodegeneration	Joanna Wardlaw, University of Edinburgh, Edinburgh, UK Sponsor country: Canada	Eleven

**Figure 1:**
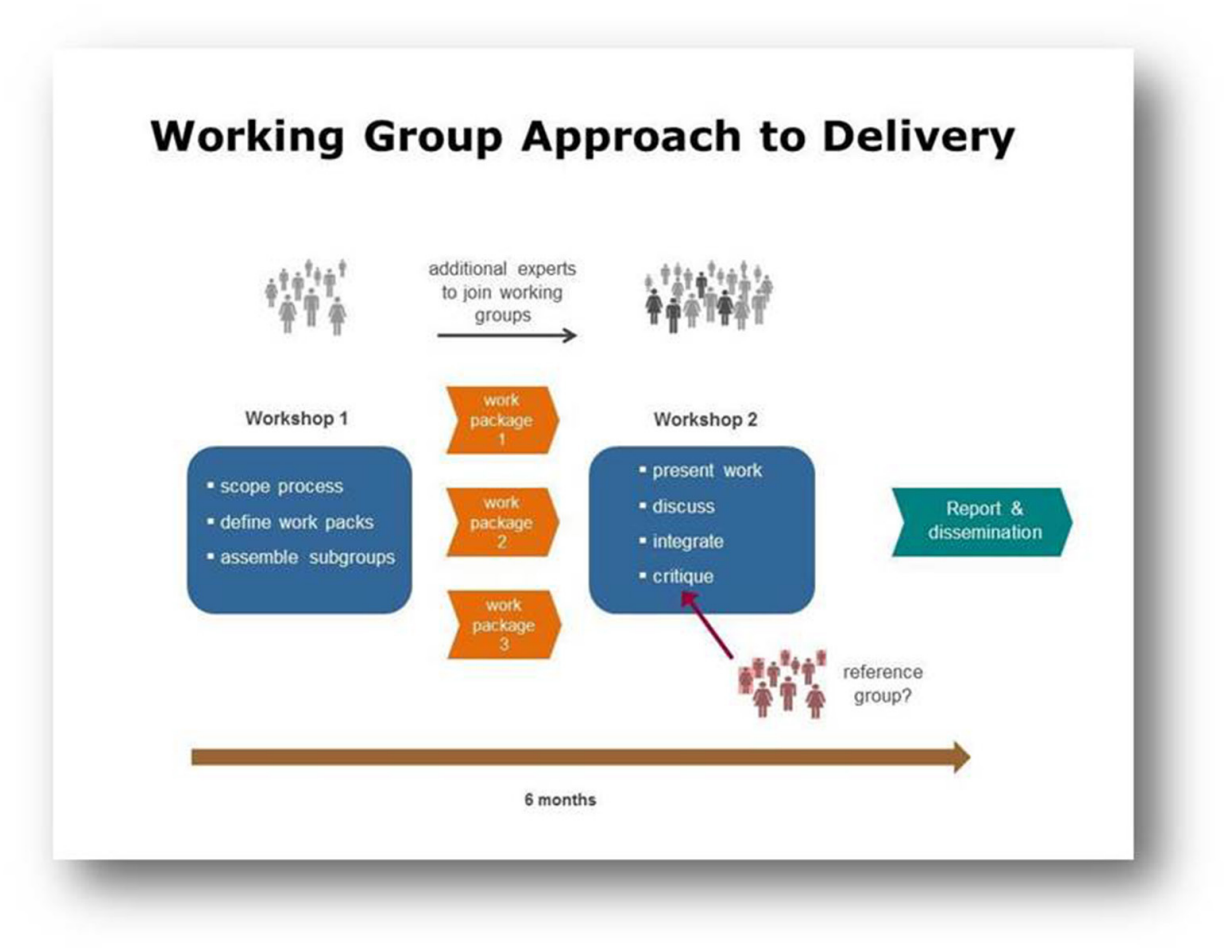
This diagram illustrates the Working Group approach to delivery through workshops, other communications and a final report.

Working Groups typically brought together 10–20 leading experts to address a specific topic area, and were asked to hold two workshops over a 6-month period, communicating by tele- or video conference in the time between meetings (Figure 1). The incorporation of external reference groups was encouraged, to ensure both objectivity of outputs and that they would be of the most use to the wider research community.

The first awards were made in August 2014, just 4 months after the launch of the call, with the final reports for the ten Working Groups received by June 2015, followed by their publication on the JPND website in October 2015^2^.

## Results

The ten Working Groups assembled under this funding action produced wide-ranging work across a broad spectrum of ND research. Some examples of their accomplishments are highlighted below.

### Realizing the potential of cohort studies to determine vascular contributions to neurodegeneration

It is estimated that a third of all dementias are due wholly or in part to vascular disease. Although vascular risk is modifiable, little is currently understood about the vascular contribution to neurodegeneration or how to treat it. The objective of the METACOHORTS Working Group was to elucidate how cohort studies might be better leveraged to investigate these links.

Led by Joanna Wardlaw from the University of Edinburgh, METACOHORTS brought together 55 international experts on brain disease and dementia to survey data from more than 90 studies, representing more than 660,000 participants. The researchers uncovered a tremendous amount of new information from existing studies that had little overlap with any previous data collections, suggesting that the value of mining such data can be very high (Dichgans et al., 2016). This Working Group subsequently secured EU Horizon 2020 funding to examine mechanisms of microvascular disease as a common cause of stroke and dementia, based on multi-center studies. Future impact of the work is expected to include the integration of stroke and dementia prevention clinics; research in AD that better assesses vascular risk factors; and research in stroke that routinely assesses cognition as well as physical outcomes.

### Dementia outcome measures: charting new psychosocial territory

In this Working Group, Gail Mountain of the University of Sheffield coordinated a group of experts focused on identifying the best outcome measures for the assessment of psychosocial interventions in dementia research. Starting from the premise that enabling people to live well with a dementia diagnosis is a key research objective, the group aimed both to develop recommendations regarding the use of existing outcome measures and to investigate the need for new measures.

When surveying the existing measures commonly used for people with dementia and their supporters, the group identified several challenges. For example, they noted that “no single theory currently provides an adequate basis for defining wellbeing in dementia,” with much of the literature rooted in a “medical loss/deficit paradigm of dementia care”^3^. Moreover, they found that the majority of existing measures do not enable people with dementia to report data on their own behalf, which can be problematic in light of research showing that proxy ratings (usually from careers) often conflict with those offered by the people living with dementia themselves^4^. The group shortlisted 33 measures for psychosocial intervention research, 16 of which were ultimately strongly recommended to the research community. The group also made a series of recommendations for novel measures. For example, the group determined that new measures not predicated upon recall should be developed. Further recommendations called for the establishment of measures that could be self-completed by people with early or moderate stages of dementia, and for the use of special technology and visual methods for people at more advanced stages. The outputs of this group, which resulted from four face-to-face workshops, a year of desk-based work, and a consultation with people living with dementia, aim to better facilitate the collation of data from cohorts of people with dementia who participate in psychosocial research across different countries. This paves the way for data sharing, ultimately helping to shape the remit and quality of future psychosocial research across the dementia trajectory^5^.

### High dimensional research in Alzheimer's Disease (HD-READy)

A cornerstone of ND research has always been the use of state-of-the-art technologies and especially imaging and genetics. Thus far, these two fields have mostly operated independently, yet many methodological considerations overlap. Both are high-throughput technologies requiring specific analytical strategies. For instance, genome-wide association studies are hypothesis-free screens of the entire genome for subtle signals involved in ND; similarly, voxel-based approaches interrogate the entire brain without assuming any pre-knowledge for regions affected in ND. In the coming years, these two fields will move closer together, opening up potentially novel avenues for research, but also bringing a new set of challenges, which the HD-READy Working Group, coordinated by Arfan Ikram of the Erasmus University Medical Centre, set out to address.

The starting point revolved around the question of how to approach a voxel-based, genome-wide association study in terms of the methodological, analytical, statistical, and computational challenges to overcome and perform, for each single voxel in the brain (*n* = 1,000,000), an entire genomic screen (*n* = 10,000,000). Using simulation and real-world datasets, the group published a set of recommendations and developed a novel analytical strategy (Adams et al., 2016; Roshchupkin et al., 2016) that can aid ND research aiming to combine imaging and genetics. The recommendations are based on four HD-READy core principles: setting (population-based vs. clinical), speed, sharing and statistics. International consortia, such as CHARGE (Psaty and Sitlani, 2013) and ENIGMA (Thompson et al., 2014), are already incorporating these recommendations in their workflows with respect to imaging and genetics. Importantly, this novel methodology can also easily be applied to other hypothesis-free, omics-technologies^6^.

### Multi-center cohort studies in dementia with lewy bodies: harmonizing clinical and biomarker protocols

This Working Group, coordinated by Dag Aarsland of Stavanger University Hospital, focused on the use of cohort studies in research on dementia with Lewy bodies (DLB). Few longitudinal studies currently exist and very little is known about the prodromal phases of DLB—information that is crucial for both clinical management and research. This group facilitated the creation of a new European DLB consortium, which has rapidly grown and collected retrospective data on >1,200 DLB patients. Recommendations were made on how best to combine data from different existing cohorts with different protocols and to pave the way for future cohort studies^7^. The consortium also serves as a trial-ready cohort and study leaders have been engaged in discussions with companies aiming to carry out trials in DLB. Finally, new funding is currently being sought for a prospective study (Biundo et al., 2016).

### Developing a methodological framework for trials in pre-symptomatic ND

Multi-center pre-symptomatic trials aim to characterize an ND from its earliest stages, establish disease biomarkers, and develop cohorts large enough for clinical trials. Yet pre-symptomatic trials for ND face a range of complex design and ethical challenges. The Pre-symptomatic Neurodegeneration Initiative (PreNI) Working Group, led by Jonathan Rohrer of the University College London Dementia Research Centre, cataloged these crucial challenges and drafted a set of guidelines for improved planning of future trials.

The group issued a set of 14 recommendations covering trial design, inclusion criteria, and the use of biomarkers. Since measures such as time-to-symptoms can lead to lengthy clinical trials, the group advocated for the need for shorter trials, highlighting the need for “proximity markers,” i.e., markers that identify a period in proximity to symptom onset. Furthermore, the group supported the use of adaptive trial design in order to increase efficiency and allow for the testing of more drugs over a shorter period. With regard to inclusion criteria, the group recommended selecting subjects for participation as early as possible, before significant irreversible neuronal loss occurs, which would require the development of imaging and fluid biomarkers for early-phase trials. The group also suggested an approach to a range of ethical issues, such as the possibility of accidental unblinding of genetic status to trial participants or the revealing of disease status in pre-symptomatic studies of sporadic disorders. The work is expected to inform the design of future drug trials in pre-symptomatic disease, and particularly to lead to the earlier consideration of ethical issues^8^.

## Discussion

The trans-national Working Groups mobilized under this funding action brought together scientists from across the globe to address methodological challenges that cannot, by definition, be solved within the borders of any one country alone. The JPND mechanism allowed groups to rapidly and efficiently provide toolkits to assist scientists in their research planning, both to unlock potential in existing studies and to help support new research. Indeed there has already been some evidence of this, with the funding of a proposal linked to the PreNI Working Group under the JPND-European Commission co-funded call, as well as the success of the METACOHORTS Working Group in securing Horizon 2020 funding^9^. The BRIDGET project, funded in 2015 by JPND, will further expand the work done by HD-READy^10^. More broadly, the connections made between group members have in many cases continued beyond the funding period, resulting in a rich stream of scientific publications.

In addition, there is evidence that the Working Groups have stimulated new ideas. One example comes from the group focused on harmonization and innovation of cognitive, behavioral and functional assessment in dementia, which was led by Alberto Costa of the IRCCS Fondazione Santa Lucia and Niccolò Cusano University^11^. This group found that the knowledge presently available on the validity, reliability and accessibility of new tools for the evaluation of cognitive disorders was limited. The goals identified by the Working Group were to achieve a shared standard set of high sensitivity and specificity tools for the pre-symptomatic stages of AD, the measurement of change during longitudinal studies, and the assessment of functional abilities.

Moreover, the systematic review of all data on the reliability of image acquisition methods for vascular disease undertaken by the METACOHORTS group provides essential underpinning for future standardization work (De Guio et al., 2016). This Working Group also recognized that cerebral microvascular disease has local and global effects on the brain, making the burden of brain damage far greater than any individual focal neurological symptom or visible lesion on a scan. As a consequence of this observation, continuing efforts are being directed at assessing variability of vascular risk vs. cerebrovascular disease burden across different cohorts, examining patterns of accumulating brain damage and mechanisms, and improving clinical trial methodologies.

The Working Groups continue to collaborate and share information in various ways. Building on their previous work, the PreNI group organized a successful international conference on pre-symptomatic ND in London (convened by Lancet Neurology, 19–21 October 2016). Several other groups are also working on new projects that extend their work. One example is BioLoC-PD, a Working Group led by Daniela Berg of the Hertie-Institute for Clinical Brain Research that focused on the harmonization of biomarker assessment in longitudinal cohort studies in Parkinson's disease (PD)^12^. After the group established the enormous potential for future joint data analyses, members of BioLoC-PD formed a partnership for data sharing, leading to biomarker analyses with much larger and statistically robust sample sizes, as well as to the replication of biomarker data in the prodromal phase of PD (Lerche et al., 2015; Lawton et al., 2016).

Despite the progress made by the Working Groups, however, several limitations were identified. For instance, Working Groups reported that the short time frame of the project was a challenge. For this reason, future actions should extend the time frame to at least 9 months. One Working Group expressed concern that obtaining funding for the reanalysis of existing data was more challenging than for new data, suggesting that there could be increased recognition by funders of the value of data mining and reanalysis. Since the Working Groups were identified through an open call, a concern was raised that such a mechanism might be biased toward more established members of the community including researchers who run large cohort studies and may have determined the original conditions for access. The authors acknowledge the importance of this point, but also maintain that potential for conflict of interest could also exist in any other group set up to examine these issues. Moreover, the policy of most funders—including JPND—is now to require researchers to make their data openly available for use by other groups. To help ensure effectiveness, funders should require custodians to operate transparent access mechanisms and have independent representation on committees responsible for deciding cohort access for research purposes. By facilitating a face-to-face policy development meeting around these issues away from the individual researchers' own laboratories, the aim of JPND was to kick-start progress toward, and lay the groundwork for, the future provision of meaningful new community standards. Finally, several Working Groups noted previous difficulty in communicating their outputs and persuading the research community to adopt the recommended measures. This paper was written partly to address this need.

## Conclusions

Research solutions are urgently needed for ND, which are associated with a huge social and economic burden, affecting not only patients but those who care for them, and numbers of patients worldwide are set to grow exponentially in coming years. Cross-border, collaborative research will play a vital role in generating future knowledge and understanding of human health and disease, as well as in the development of safe and effective treatments.

Sometimes in research, enabling steps are first required before substantive studies can accelerate the pace of research progress. In the case of the ND field it is well-recognized that there is an urgent need to understand disease onset and progression as a basis for future interventions, and in principle, longitudinal cohort studies offer an excellent approach because they provide a wealth of information available over time. Yet the use of cohort studies in ND research has not been as prevalent as expected, primarily due to very practical considerations such as lack of knowledge about which cohorts are available and suitable for what purposes, as well as more fundamental methodological challenges, such as variations in data collection and measurement.

The rapid, flexible funding mechanism leveraged in this action represents a new cross-border funding tool that has the potential to help accelerate scientific research rapidly and at relatively low cost. This flexible funding approach is now being extended to other JPND priority areas, with JPND again implementing the mechanism in its 2016 call for Working Groups for harmonization and alignment in brain imaging, which draws on experience and expands the duration of the award to 9 months.

The JPND Longitudinal Cohort Studies Working Groups brought together some 200 leading experts to build consensus around key challenges inhibiting research progress. The collective outputs of the Working Groups demonstrate that the “rapid action” approach described here has proved efficient and effective, resulting in the stimulation of novel ideas, the formation of new partnerships for future research or data sharing and the development of best-practice frameworks that now form the essential underpinnings for future standardization work.

## Author contributions

DM and CM conceived the article and participated in its coordination. DM and CM drafted the manuscript with assistance from all authors and GK assisted considerably with the revision. Information on each WG was submitted for consideration by an individual author, with assistance from those acknowledged below in the “JPND Longitudinal Cohort Studies WG Consortium.” All co-authors read and approved the final manuscript.

### Conflict of interest statement

The authors declare that the research was conducted in the absence of any commercial or financial relationships that could be construed as a potential conflict of interest.

## Footnotes

^1^http://www.neurodegenerationresearch.eu/uploads/media/JPNDAGLCS_Final_Report_Oct_2013-version_07_01_14.pdf

^2^http://www.neurodegenerationresearch.eu/initiatives/jpnd-alignment-actions/longitudinal-cohorts/call-for-working-groups/reports

^3^http://www.neurodegenerationresearch.eu/wp-content/uploads/2015/10/JPND-Report-Fountain.pdf

^4^Ibid.

^5^Ibid.

^6^http://www.neurodegenerationresearch.eu/wp-content/uploads/2015/10/JPND-Report-Ikram.pdf

^7^http://www.neurodegenerationresearch.eu/wp-content/uploads/2015/10/JPND-Report-Aarsland.pdf

^8^http://www.neurodegenerationresearch.eu/wp-content/uploads/2015/10/JPND-Report-Rohrer.pdf

^9^http://cordis.europa.eu/project/rcn/199717_en.html

^10^http://www.neurodegenerationresearch.eu/wp-content/uploads/2015/10/Fact-Sheet_BRIDGET_v2.pdf

^11^http://www.neurodegenerationresearch.eu/wp-content/uploads/2015/10/JPND-Report-Costa.pdf

^12^http://www.neurodegenerationresearch.eu/wp-content/uploads/2015/10/JPND-Report-Berg.pdf

## References

[B1] Adams H. H. Adams H. Launer L. J. Seshadri S. Schmidt R. Bis J. C. (2016). Partial derivatives meta-analysis: pooled analyses when individual participant data cannot be shared. bioRxiv 038893 10.1101/038893

[B2] Biundo R. Weis L. Bostantjopoulou S. Stefanova E. Falup-Pecurariu C. Kramberger M. G.. (2016). MMSE and MoCA in Parkinson's disease and dementia with Lewy bodies: a multicenter 1-year follow-up study. J. Neural Transm. Vienna Austria 123, 431–438. 10.1007/s00702-016-1517-6 26852137 PMC4820017

[B3] Colonna M. Wang Y. (2016). TREM2 variants: new keys to decipher Alzheimer disease pathogenesis. Nat. Rev. Neurosci. 17, 201–207. 10.1038/nrn.2016.7 26911435

[B4] De Guio F. Jouvent E. Biessels G. J. Black S. E. Brayne C. Chen C.. (2016). Reproducibility and variability of quantitative magnetic resonance imaging markers in cerebral small vessel disease. J. Cereb. Blood Flow Metab. 36, 1319–1337. 10.1177/0271678X16647396 27170700 PMC4976752

[B5] Dichgans M. Wardlaw J. Smith E. Zietemann V. Seshadri S. Sachdev P. (2016). METACOHORTS for the study of vascular disease and its contribution to cognitive decline and neurodegeneration: an initiative of the joint programme for neurodegenerative disease research. Alzheimers Dement. 12, 1235–1249. 10.1016/j.jalz.2016.06.004 27490018 PMC5399602

[B6] Goedert M. (2015). NEURODEGENERATION. Alzheimer's and Parkinson's diseases: the prion concept in relation to assembled Aβ, tau, and α-synuclein. Science 349:1255555. 10.1126/science.1255555 26250687

[B7] Lawton M. Kasten M. May M. T. Mollenhauer B. Schaumburg M. Liepelt-Scarfone I.. (2016). Validation of conversion between mini-mental state examination and montreal cognitive assessment. Mov. Disord. 31, 593–596. 10.1002/mds.26498 26861697 PMC4864892

[B8] Lerche S. Liepelt-Scarfone I. Alves G. Barone P. Behnke S. Ben-Shlomo Y.. (2015). Methods in neuroepidemiology characterization of european longitudinal cohort studies in parkinson's disease–report of the JPND working group BioLoC-PD. Neuroepidemiology 45, 282–297. 10.1159/000439221 26523894

[B9] Psaty B. M. Sitlani C. (2013). The cohorts for heart and aging research in genomic epidemiology (CHARGE) consortium as a model of collaborative science. Epidemiology 24, 346–348. 10.1097/EDE.0b013e31828b2cbb 23549178

[B10] Rohrer J. D. Isaacs A. M. Mizielinska S. Mead S. Lashley T. Wray S.. (2015). C9orf72 expansions in frontotemporal dementia and amyotrophic lateral sclerosis. Lancet Neurol. 14, 291–301. 10.1016/S1474-4422(14)70233-9 25638642

[B11] Roshchupkin G. V. Adams H. H. H. Vernooij M. W. Hofman A. Duijn C. M. V. Ikram M. A.. (2016). HASE: framework for efficient high-dimensional association analyses. Sci. Rep. 6:36076. 10.1038/srep36076 27782180 PMC5080584

[B12] Thompson P. M. Stein J. L. Medland S. E. Hibar D. P. Vasquez A. A. Renteria M. E.. (2014). The ENIGMA Consortium: large-scale collaborative analyses of neuroimaging and genetic data. Brain Imaging Behav. 8, 153–182. 10.1007/s11682-013-9269-5 24399358 PMC4008818

